# Using normalisation process theory to evaluate the implementation of a complex intervention to embed the surgical safety checklist

**DOI:** 10.1186/s12913-018-2973-5

**Published:** 2018-03-09

**Authors:** Brigid M. Gillespie, Emma Harbeck, Joanne Lavin, Therese Gardiner, Teresa K. Withers, Andrea P. Marshall

**Affiliations:** 1 School of Nursing & Midwifery, Griffith University, Gold Coast, QLD Australia; 2 Gold Coast Hospital and Health Service, Gold Coast, QLD Australia; 3 National Centre of Research Excellence in Nursing, Menzies Health Institute of Queensland, Griffith University, Gold Coast, QLD Australia; 4 Surgical and Procedural Services, Gold Coast Hospital and Health Service, Gold Coast, QLD Australia; 5 Nursing & Midwifery Education & Research Unit, Gold Coast Hospital and Health Service, Gold Coast, QLD Australia

**Keywords:** Surgical safety checklist, Implementation, Evaluation, Tailoring, Sustainment, NoMAD tool, Pass the baton, Operating room

## Abstract

**Background:**

The surgical Safety Checklist (SSC) was introduced in 2008 to improve teamwork and reduce the mortality and morbidity associated with surgery. Although mandated in many health care institutions around the world, challenges in implementation of the SSC continue.

To use *Normalisation Process Theory* (NPT) to help understand ho*w*/why implementation of a complex intervention coined *Pass The Baton (PTB)* could help explain what facets of the Surgical Safety Checklist use led to its’ integration in practice, while others were not.

**Methods:**

A longitudinal multi-method study using survey and interviews was undertaken. Implementation of *PTB* involved; change champions, audit and feedback, education and prompts. Following implementation, surgical teams were surveyed using the NOrmalization MeAsure Development (NoMAD) and subsequently interviewed to explore the impact of *PTB* on their use of the checklist at 6 and 12 months respectively. Respondents’ self-reported perceptions of implementation of *PTB* was explained using the four NPT constructs; *coherence, cognitive participation, collective action*, and *reflexive monitoring*. Survey data were analysed using descriptive statistics. Interview data were coded inductively and content analysed using a framework derived from NPT.

**Results:**

The NoMAD survey response rate was 59/150 (39.3%). Many (45/59, 77.6%) survey respondents saw the value in *PTB*, while 50/59 (86.2%) would continue to use it; 45/59 (77.6%) believed that *PTB* could easily be integrated into existing workflows, and 48/59 (82.8%) thought that feedback could improve *PTB* in the future.

A total of 8 interviews were completed with 26 surgical team members. Nurses and physicians held mixed views towards coherence while buy-in and participation relied on individuals’ investment in the implementation process and the ability to modify *PTB*. Participants generally recognised the benefit and value of using *PTB* in the ongoing implementation the checklist.

**Conclusions:**

Workarounds and flexible co-construction in implementation designed to improve team communications in surgery may facilitate their normalisation in practice.

**Electronic supplementary material:**

The online version of this article (10.1186/s12913-018-2973-5) contains supplementary material, which is available to authorized users.

## Background

In 2008, the World Health Organisation World Alliance launched the “Safe Surgery Saves Lives” campaign and developed the Surgical Safety Checklist (SSC) to enhance intraoperative teamwork and thus, minimise the risk of intraoperative adverse events and postoperative complications [1, 2]. The general purpose of the SSC is to ensure the performance of essential preoperative procedures, resolve questions, address team concerns and provide fail-safe confirmation about procedure-specific information. The SSC has three checkpoints containing items specific to each checkpoint; 1) before anaesthetic induction (sign-in); 2) before surgical incision (time-out), and 3) before the patient leaves the operating room (sign-out) [1]. Yet despite the encouraging results of many published studies [3–5] including several meta-analyses that suggest use of the SSC leads to reductions in patient morbidity and mortality [6, 7], compliance and sustained use remains a challenge [4, 8].

This challenge arises from differing institutional contexts with inconsistent implementation strategies. Other factors such as professional hierarchies, excessive documentation requirements, increased subspecialisation, lack of interdisciplinary collaboration, and a general scepticism of any additional intraoperative interventions [8, 9]. These factors have led to variations in practice. Although variation in SSC execution was intended to encourage hospitals and health services to tailor the checklist to their specific needs [8], this has often created tensions between universal best practice mandates and the unique local context of a particular institution [10]. Understanding the complexity associated with the implementation of any multifaceted intervention entails consideration of diverse delivery conditions including the individuals and collectives of people tasked with ‘normalising’ it as part of everyday practice in the context of providing safe care. Thus, using theory-led research designs to explain the implementation and integration of multifaceted interventions may inform the development of strategies to embed their use in practice.

### Normalisation process theory

May and Finche’s [11] Normalisation Process Theory (NPT) is concerned with the social organisation of work (implementation), of routinizing elements of practice into everyday life (embedding), and of sustaining (integrating) embedded practices in their social contexts. Based on the propositions that underscore NPT [11, 12] an intervention that gets embedded in practice is likely to be one that allows flexible achievement of both congruence and disposal. Flexibility is required for all stakeholders to associate their ideas and beliefs, (i.e., congruence) and operationalise them in outcomes that are meaningful (i.e., disposal) [11]. The second process within NPT is relational integration [12]. Accordingly, this network of relations is how the knowledge and practice of the intervention is defined and mediated over time. This incorporates two dimensions, *accountability* and *confidence* [12]. *Accountability* refers to internal network and has three components. These include: i) validity of the knowledge associated with the intervention which encompasses the ways in which disputes about that knowledge are minimised and the distribution of knowledge within the hierarchies of the network; ii) expertise, beliefs about the expertise entailed in the intervention; and iii) dispersal, the distribution of knowledge and practice within the network. *Confidence* refers to the external network and has three components [12]. These are: i) credibility, i.e., achieving a shared understanding of the credibility of the intervention and the ways in which disagreements about the intervention are managed; ii) agreement about how the credibility of the intervention is measured; and, iii) utility, beliefs about the source of knowledge and the expectations about the authority of the dispersion of knowledge to external network.

Constructs of NPT include*; coherence or sense-making, cognitive participation, collective action,* and *reflexive monitoring* [12]. *Coherence* refers to the sense-making work that people do individually and collectively when they are faced with implementing a set of practices. *Cognitive participati*on refers to the relational work that people do to build and sustain a community of practice around a complex intervention or new technology. *Collective action* refers to the operational work that people do to enact a set of practices, whether these represent a complex intervention or new technology. *Reflexive monitoring* refers to the appraisal work that people do to assess and understand that ways in which a new set of practices affect them and others around them.

As a middle range theory, NPT has been increasingly applied to many different healthcare specialities and contexts, including mental health [13], allied health [14], acute healthcare [15], primary healthcare settings [16, 17] and medical revalidation [18] as an explanatory model to guide the development and implementation of complex interventions. We chose the NPT as an explanatory model to explore and evaluate the implementation of a complex intervention in the Operating Room (OR) context. The aim of our study was to describe participants’ views about how the implementation of a complex intervention coined *Pass The Baton (PTB)* influenced their use of the Surgical Safety Checklist (SSC), and their expectations about whether the SSC could become routinized in their work. To this end, we used the NPT and the four constructs *of coherence cognitive participation, collective action* and *reflexive monitoring* to understand what aspects of the SSC use led to it being *normalised* in practice, while other aspects of its use were not.

## Methods

### Study overview

To evaluate the embedding and sustainment of a complex intervention to promote checklist compliance, we used a longitudinal multi-method design that included a survey at 6 months followed by individual or focus group interviews at 12 months post-implementation of the *Pass The Baton (PTB)* intervention. The *PTB* intervention was implemented over four weeks during November 2015, and survey data collected in May 2016, 6 months following its implementation. Interview data were collected in December 2016, 12 months’ post-implementation.

### Pass the baton (PTB) intervention

Although a modified version of the SSC containing 20 items across the Sign-in, Time-out and Sign-out phases was mandated in the study hospital in 2012, its implementation throughout the department was inconsistent and fragmented. A barriers analysis based on the findings of an observational audit and 33 semi-structured interviews with 70 stakeholder participants indicated that the most significant barriers to sustained checklist implementation were; workflow, a lack of knowledge about content/timing of the checks, a lack of clinical leadership, and dissenting attitudes towards the benefit of checklists in surgery [19, 20]. To address these barriers, the *PTB* intervention was delivered over four weeks and included several strategies designed to promote behaviour change. As part of the implementation process, development of the program was co-constructed with end-users who included operating room nurses working in circulating/instrument and anaesthetic roles and physicians specialising in anaesthetics and surgery. The implementation team included a senior surgeon, staff nurses working in education, circulating/instrument and anaesthetic roles, and researchers with expertise in knowledge translation. Coproduction employed the distinct expertise of clinical and academic members of the implementation team: The clinical team brought expertise in clinical practice and process, while the academic team brought expertise in intervention development, interpretation and evaluation based on rigorous methods. As the development and implementation of the intervention was driven by key clinical stakeholders, it was more likely to be embedded readily into clinical practice and sustained over time [21, 22].

**Fig. 1:**
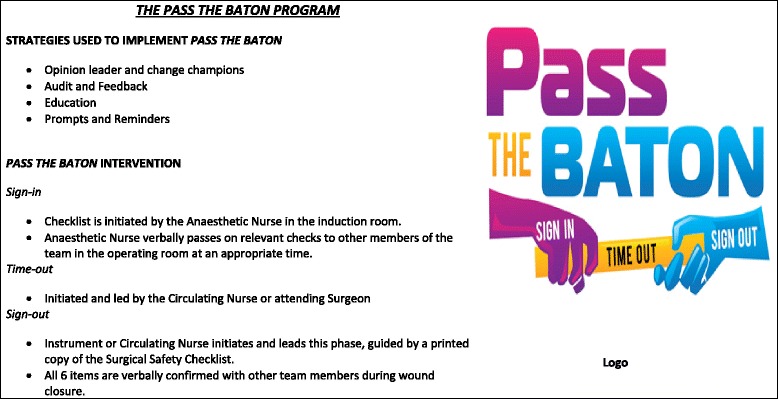
Implementation strategies used to support *Pass The Baton* and a description of the *Pass The Baton* Intervention

The *PTB* intervention focused on designating a team member, usually the Circulating nurse or Surgeon, to lead the deliberate communication of case-related information to other members of the team. Due to workflow, it was often unfeasible for members of the anaesthetic, surgical and nursing teams to participate as a collective during all phases of checklist execution [20]. Prior to *PTB*, Sign-in checks were undertaken by both the circulating and anaesthetic nurses at different times while the patient was in the induction room. When workflow allowed, the anaesthetist would also perform some checks. Timeout and Sign-out were undertaken in the OR, so essentially all members were in the one place. However, while Timeout items reached 70% compliance, Sign-out was not observed at all prior to implementation of *PTB*. Overall checking processes were fragmented relative to *who* did *what* checks, and *how* and *when* these checks were communicated to others. Using *PTB* as a workaround, the anaesthetic nurse would undertake the safety checks during Sign-in and subsequently communicate case-relevant information to other team members, who were in the OR. The Circulating nurse or Surgeon was responsible for leading Timeout while the Circulating nurse led Sign-out. The *PTB* the intervention and the strategies used to support its implementation are summarised in Fig. 1.

### Setting and sample

This study was conducted at a 750 bed university hospital in Queensland Australia. At the time of this study, the operating room department employed 150 medical, nursing and 50 operational (i.e., porters and administration officers) staff, had 22 commissioned theatres, and performed 16,000 surgeries per year in all specialties except transplantation.

Inclusion as a study participant for both the survey and the interviews required prior exposure to the implementation of the *PTB* intervention. Our aim was to recruit those actors who were closely involved in the development and design of the intervention, as well as those participants who were end-users of the intervention. The sampling frame included a total of 150 professional staff practising as consultant and trainee surgeons and anaesthetists, and circulating, instrument and anaesthetic assistant nurses. Thus for the survey, a convenience sample of these staff was invited to participate. Interview participants were drawn from the same sample and included a purposive subsample of those staff.

### Survey tool and data collection

#### Survey

The recently developed NOrmalization MeAsure Development (NoMAD) tool, which consists of 23 items: 20 items that reflect the four NPT constructs (coherence-4 items, *cognitive participation-*4 items*, collective action-*7 items and *reflexive monitoring-*5 items) and 3 items that provide a general assessment of respondents’ expectations and experience of the intervention’s implementation process [23], was used. Therefore, this instrument measures the implementation processes from the perspective of professionals directly involved in the work of implementing complex interventions. Development of the initial 46 item instrument included several iterative rounds of item generation, consensus, workshops, “think aloud” interviews, item quality appraisal and theory validation [23, 24]. The authors report that the final 23 items was the result of psychometric analysis and the examination of response patterns from six different implementation projects, providing an overall sample of 831 respondents for validation purposes [24–26].

Since its development, the NoMAD tool is gaining popularity among researchers. Sturgiiss et al*’s*. [17] multi-methods study used the NoMAD tool to evaluate the feasibility of implementing a weight management program from a general practitioner’s (GP) perspective. With only a sample of 10 GPs surveyed, the primary results focused on the level and frequency of agreement. Hazell et al*’s* [13] methodological study in a sample of 201 mental health clinicians used exploratory factor analysis (EFA). Results indicated the NoMAD tool had a three-factor structure: *coherence* (8 items) *cognitive participation* (6 items) and *reflexive monitoring* (6 items). *Collective action* items were within these three factors. All three scales demonstrated strong internal consistency, with the lowest Cronbach’s alpha α = 0.79 [13].

We chose the NoMAD tool because it is the first quantitative measure based on the four constructs of NPT and was suitably vague to enable use across various settings. We customised the NoMAD to meet the needs of our target respondents and the intervention being implemented (see Additional file 1), as suggested by its creators [23, 26]. The NoMAD tool allows comparisons across the four NPT constructs at the item (*n* = 20) level and the factor level, using a 5–point Likert scales to indicate the level of agreement, where 1 = *strongly agree*, 3 = *neutral* and 5 = *strongly disagree*. The three general questions about the intervention from the NoMAD are rated using a response scale of 0–10 where 0 = *not at all,* 5 = somewhat and 10 = *completely* [23, 24]. Several members of the implementation team distributed the paper-based NoMADS survey to medical and nursing staff during staff meetings and inservices.

#### Interviews

Semi-structured face-to-face individual and focus group interviews with a subsample of survey respondents were performed by the lead author, lasted between 20 and 40 min, and were digitally recorded and transcribed. Access to staff for interviews usually occurred during quieter periods, when there was limited surgical activity. The type of interview method was determined by staff availability and interviews were conducted at the participant’s convenience, in a quiet area away from the clinical environment. Group interviews were conducted with nurse participants who belonged to the same staff category to ensure group homogeneity, and minimise the potential impact of hierarchy on group dynamics [27].

An interview guide divided into themes based on the four constructs of NPT allowed exploration of participants’ experiences of implementing *PTB* was used. During interviews, participants were asked to describe their involvement in PTB and to share their perspectives about the most significant change that had occurred in using the checklist, and how, if at all, *PTB* had contributed to that process. Additionally, participants were asked about the extent to which they had adapted the *PTB* and if not, why not. Participants were also asked whether any further strategies were introduced to support or sustain *PTB*, and which of the implementation strategies contributed most to any change in checklist use. Participants were encouraged to raise any issues they thought relevant, and the interviewer used methods such as probing to help achieve depth. Additional file 2 provides examples of the interview questions we asked.

### Ethics approval

Ethics approval was provided by the hospital and the university Human Research Ethics Committees (HREC/13/QGC/154 and NRS/06/14/HREC respectively). For the survey, completion and return of the survey form indicated informed consent. Prior to interview participants signed a consent form to indicate informed consent. This confirmed that participants understood that their participation was voluntary and that they were willing to allow the lead author include anonymous quotations from them in the write up of the study.

### Analysis

#### Quantitative data

The NoMAD tool [23, 26] was analysed descriptively using absolute (*n*) and relative frequencies, and means, standard deviations (SD) as appropriate to the level of the data and 95% confidence intervals (CI) were used. For analysis and interpretation, the Likert response format was recoded to 1 = *strongly disagree* to 5 = *strongly agree,* so higher item and factor mean scores indicated higher agreement from our sample. One item from the Collective action factor (*Pass the Baton disrupts working relationships*) was reversed scored due to its negative valence. In line with the guidance provided by the tool’s creators [23, 24], total scores for the NoMAD were not calculated. Internal consistency of the four NoMAD factors was examined using Cronbach’s alpha (α). Alpha’s ≥ .70 indicate acceptable levels of reliability [28].

#### Qualitative data

We used inductive content analysis as described by Elo and Kyngäs [29] to code interview transcripts. These codes were subsequently collapsed into subcategories using a framework approach, mapping against the core constructs and components of NPT [11, 26]. Descriptive codes were generated and organised through content analysis under the framework based on the four constructs of NPT. Preliminary categories were identified using this framework and were compared across the data. For example, under the construct *coherence*, we coded data in relation to the components of ‘differentiation’ (*How is PTB different from what you are already doing when using the checklist?*), ‘communal specification’ (*Do members of the surgical team agree on the intent and benefit of using PTB?*), ‘individual specification’ (*Do team members understand how PTB affects their day-to-day roles, tasks and responsibilities?*), and ‘internalisation’ (*Is there coherence in the tasks and responsibilities that comprise PTB?*). We assessed the textual data for disparities to ensure that we did not discount issues that did not readily onto the NPT framework. The research team held regular meetings during the analytic process to ensure rigour in the analytic process, and decisions reached by consensus.

## Results

### Survey

Staff participation in the survey was 39.3% (59/150). Table 1 shows the demographic characteristics of survey respondents. Most (50/59, 84.4%) survey respondents were female. Using the NoMAD general questions about the intervention, overall 63.8% (*n* = 58) of participants rated *PTB* as familiar (M = 6.3, SD = 2.7). While *PTB* was implemented department-wide, 59.6% (*n* = 57) indicated they thought that *PTB* was currently a normal part of their work (M = 6.5, SD = 2.5) and 76.8% (*n* = 56) indicated they believed *PTB* will become a normal part of their work (M = 7.8, SD = 2.1). In terms of *coherence*, the majority (*n* = 45, 77.6%) of survey respondents agreed that ‘*PTB* was of value in their work’. In relation to *cognitive participation*, most (*n* = 50, 86.2%) respondents indicated that they would ‘support *PTB* in the future’. Insofar as *collective action*, the majority (*n* = 45, 77.6%) of respondents agreed that ‘*PTB* could be easily integrated into their existing work’. With regard to *reflexive monitoring*, most (*n* = 48, 82.8%) respondents agreed that ‘their feedback could be used to improve *PTB* in the future’. Table 2 shows the descriptive results for the NoMAD survey tool. Total mean scores were highest in relation to the construct, *cognitive participation* (M = 16.0, SD = 2.0). Cronbach’s alpha ranged from α 0.68─α 0.74 across the four NPT constructs.

**Table 1:** Surveyed participants’ demographic characteristics (*n* = 59)

	Nurse	Doctor	Total
Sampled *n* (%)	48 (81.4)	11 (18.6)	59
Gender *n* (%)			
Female	45 (93.8)	5 (45.5)	50 (84.7)
Male	3 (6.3)	6 (54.5)	9 (15.3)
Primarily employed as *n* (%)			
Enrolled nurse	4 (8.4)	–	4 (6.8)
Registered Nurse	35 (72.9)	–	35 (59.3)
Clinical nurse	9 (18.8)	–	9 (15.3)
Registrar	–	6 (54.5)	6 (10.2)
Consultant	–	5 (45.5)	5 (8.5)
Years of experience in current clinical role^a^			
M (SD)	9.2 (9.9)	5.4 (4.7)	8.5 (9.3)
Qualification *n* (%)^a^			
Degree (includes diploma, bachelor and graduate)	36 (75.0)	6 (54.5)	42 (71.2)
Postgraduate degree (includes Masters and PhD)	11 (22.9)	5 (45.5)	16 (27.1)
Speciality *n* (%)^a^			
Anaesthetics	24 (50.0)	11 (100.0)	35 (59.3)
Surgery	24 (50.0)	0 (0.0)	24 (40.7)

^a^Missing data

**Table 2:** Normalisation Process Theory frequencies of agreement, total scores, and reliability coefficients (*n* = 59)

Item	Mean	SD	Agree n (%)	Disagree n (%)	Neutral n (%)
Sense Making (coherence)					
I can see how pass the baton differs from usual ways of working	3.3	0.9	29 (50.0)	10 (17.2)	18 (31.0)
Staff in this organisation have shared understanding of the purpose of pass the baton	3.6	0.9	36 (62.1)	8 (13.8)	13 (22.4)
I understand how pass the baton affects the nature of my own work	3.8	0.7	44 (75.9)	3 (5.2)	10 (17.2)
I can see the value of pass the baton for my work	3.9	0.8	45 (77.6)	3 (5.2)	9 (15.5)
Cognitive participation					
There are key people who drive pass the baton forward and get others involved	3.8	1.0	42 (72.4)	7 (12.1)	6 (10.3)
I believe that participating in pass the baton is a legitimate part of my role	4.1	0.7	48 (82.8)	1 (1.7)	7 (12.1)
I am open to working with colleagues in new ways to use pass the baton	4.1	0.5	49 (84.5)	0 (0.0)	6 (10.3)
I will continue to support pass the baton	4.1	0.5	50 (86.2)	0 (0.0)	6 (10.3)
Collective Action					
I can easily integrate Pass The Baton into my existing work	3.9	0.7	45 (77.6)	1 (1.7)	10 (17.2)
^a^Pass The Baton disrupts working relationships	3.8	0.9	4 (6.9)	36 (62.1)	15 (25.9)
I have confidence in other people’s ability to use Pass The Baton	3.5	0.8	33 (56.9)	6 (10.3)	17 (29.3)
Work is assigned to those with skills appropriate to Pass The Baton	3.1	0.9	18 (31.0)	8 (13.8)	30 (51.7)
Sufficient training is provided to enable staff to implement Pass The Baton	3.4	0.9	29 (50.0)	10 (17.2)	17 (29.3)
Sufficient resources are available to support Pass The Baton	3.6	0.8	34 (58.6)	6 (10.3)	16 (27.6)
Management adequately supports Pass The Baton	3.7	0.8	36 (62.1)	4 (6.9)	16 (27.6)
Reflexive Monitoring					
I am aware of reports about the effects of Pass The Baton	3.1	1.1	21 (36.2)	15 (25.9)	22 (37.9)
The staff agree that Pass The Baton is worthwhile	3.4	0.8	27 (46.6)	6 (10.3)	25 (43.1)
I value the effects that Pass The Baton has had on my work	3.7	0.6	36 (62.1)	1 (1.7)	21 (36.2)
Feedback about Pass The Baton can be used to improve it in the future	4.0	0.6	48 (82.8)	1 (1.7)	9 (15.5)
I can modify how I work with Pass The Baton	3.7	0.7	36 (62.1)	3 (5.2)	18 (31.0)
Total Scores	Mean	SD	95% CI	Range	Alpha
Sense Making (coherence) (4–20)	14.6	2.4	14.0–15.3	5–20	0.74
Cognitive Participation (4–20)	16.0	2.0	15.5–16.6	11–20	0.68
Collective Action (7–35)	25.1	3.9	24.0–26.1	14–34	0.72
Reflexive Monitoring (5–25)	17.9	2.7	17.2–18.7	12–25	0.71

^a^Item reverse scored

### Interviews

Interviews: Altogether, 8 interviews with 26 participants (5 group, 3 individual) were conducted 12 months after implementation. Participants included 3 surgeons, 2 anaesthetists and 21 nurses who worked in circulating and instrument (*n* = 15), anaesthetic (*n* = 4) and post-anaesthetic care unit (*n* = 2) roles. Table 3 details mapping of the NPT mechanisms, juxtaposed to the qualitative findings. We report qualitative findings under the four distinct, but interrelated categories:

**Table 3:** Core constructs, Generative mechanisms of NPT and illustrative questions mapped against categories and codes identified through 8 qualitative interviews (*n* = 26)

Core Constructs and Generative Mechanisms of NPT (May et al. [15])	Questions illustrative of NPT Constructs	Categories and Listed Codes
*Coherence* • Differentiation • Communal specification • Individual specification • Internalisation	1. *How is PTB understood by participants?* 2. *How do participants compare PTB to current practices when using the checklist?*	*Divergent perspectives make it challenging to achieve congruence in practice.* • Negotiating the work • Resisting change • Questioning the relevance of the process
*Cognitive Participation* • Initiation • Enrolment • Legitimation • Activation	3. *How did participants come to take part in PTB?* 4. *What keeps them motivated to continue?*	*Getting buy-in to drive participation relies on the capacity of individuals to invest in the work.* • Investing time • Modelling practice • Championing participation • Sharing expertise
*Collective Action* • Interactional workability • Relational integration • Skill set workability • Contextual integration	5. *How do participants make PTB work?* 6. *How are their activities organised and structured?*	*Modifying the work to make it more relevant and workable in practice.* • Rationalising and reorganising the work • Adapting the work to the workflow • Being consistent in the process
*Reflexive Monitoring* • Systemisation • Communal appraisal • Individual appraisal • Reconfiguration	7. *How do participants evaluate PTB?* 8. *How does PTB change over time and what are its effects?*	*Realising the benefit and reflecting on the value* • Understanding the consequences • Auditing processes • Seeing process improvements

#### Divergent perspectives make it challenging to achieve congruence in practice

The first mechanism of NPT, ‘coherence’ was one of the key processes to emerge from the qualitative data. The key question centred on *“what is the work?”* Surgical team members offered conflicting views to using *PTB* processes. While some participants appreciated the value of the checklist in enhancing team communications, others, particularly anaesthetists, resisted and questioned its relevance and the evidence upon which it was based. This was particularly so with the Sign-out process:

*How do I know whether I have any anaesthetic concerns when I have not even extubated the patient? It’s the timing; often we are asked these questions during the busiest and most vulnerable period.* (Consultant Anaesthetist, individual)

Some physicians believed the implementation of *PTB* was redundant as they were already doing these checks and found it difficult to differentiate it from other already established practices. Nonetheless over time, checklist use had improved as senior nurses persisted with the implementation of *PTB*, despite disparaging remarks made by some anaesthetists.

*‘Timeout’ took a long time to stick, but through perseverance, has become a part of our practice.* (RN, group)

Thus the active investment in, and internalisation of, checking processes contributed to end users sharing a meaning learned through embedding the checks by internalising their lived experiences.

#### Getting buy-in to drive participation relies on the capacity of individuals to invest in the work

Central to ‘cognitive participation’ is the question of *“who does the work?”* Engagement with *PTB* relied on staff to invest time in its implementation, and during this process, senior nurses initiated and modelled checking processes, particularly during the Sign-out phase:

*Consistency allows assurance to come through and that gives nurses more confidence, I have watched some do it and then others follow.* (RN, group)

The most effective way of enrolling team members was to engage them in the process of developing *PTB* and the strategies that supported it. Nurse leaders and some surgeons, when engaged in the work involved others in the process. Achieving buy-in of some professional groups often relied on nurses’ capacity to coordinate their actions in using *PTB* to undertake the checks. Senior staff championed *PTB* and provided leadership in involving others to contribute collectively to the process:

*I have kept the Checklist as a standing item on the agenda.* (Consultant Surgeon, individual)

*You just have to hand-rail them [anaesthetists] along [to do the checks] and you need a strong assertive influence to keep it going.* (RN, group)

However, legitimation of the practice was also closely bound to the norms and conventions that characterised professional identity:

*PTB has been nurse driven so not quite valued because it has to come from them [physician].* (RN, individual)

In some instances, there was a clear disconnect in the level of engagement team members demonstrated, with limited, if any ownership of the process:

*I hear some of this stuff [checks] happening, so I’m aware of it happening, but I’m not actively involved in the process…...* (Consultant Anaesthetist, individual)

#### Modifying the work to make it more relevant and workable in practice

The third mechanism, ‘collective action’ focuses on how new interventions are implemented into practice, that is, *“how does the work get done?”* Participants were most able to execute the checklist consistently when strategies such as *PTB* were tailored to fit into existing workflows and clinical routines. Additionally, to minimise the perceived impost associated with checks, nurses confirmed the checks as single word responses to minimise the cognitive load of surgeons:

*When I do the ‘Sign-out’, I ask, “Have you done an oophorectomy, left side?” I say what I think they have done, and then they can confirm it. Because the surgeon is concentrating….You don’t want them distracted, so asking “yes/no” questions is better.* (RN, group)

Simplification minimised the workload, and resulted in greater participation in the process. During implementation of *PTB*, nurses described the clear allocation of roles and responsibilities, and the ‘contextual integration’ between *PTB* and established hospital policies and procedures. Participants believed that the wordings of some checklist items, particularly during Sign-out, needed tailoring

*For Sign-out, if the wording of “are there any concerns?” were changed to “any anticipated concerns?”* (RN, group)

‘Interactional workability’ of the practice or process relies on how “actors” operationalise it. For participants, reinvention, rationalising or reorganising of the work required ongoing investment from the collective, but it was not always obvious how to coordinate their efforts to operationalise these modifications. For some, greater cognitive participation in checks, particularly in Sign-out would be achieved by revising the items to make them more meaningful to end-users:

*For the Sign-out to be relevant there needs to be a question about when the patient is going to be discharged. Having this question would be more useful and alert the staff. (*Consultant Surgeon, group)

Consistency and “perseverance” in the process to execute the checks were considered pivotal to ‘normalising’ checking processes in everyday work:

*Since PTB Sign out is verbalised, and now “you just do it” similar to Timeout.* (RN, individual)

*PTB helped at the beginning but as it has gone on people are doing it; there is no one to remind us*. (RN, individual)

Engagement with implementation of *PTB* albeit patchy, increased when senior nurses initiated and led the checks, particularly in Sign-out.

#### Realising the benefit and reflecting on the value

‘Reflexive monitoring’ of the work is essential to embedding and maintaining new clinical practices. The key question relative to appraisal is *how is the work understood?* Within this mechanism, patterns of collective action are continuously evaluated both formally and informally by participants during the implementation process. During implementation of *PTB,* checklist compliance was audited regularly, and feedback given both formally and informally to all staff. Three-monthly audit results were posted on the education boards within the department, providing staff with information about trends and illustrating differences in checklist use among surgical specialties. Interactive information sessions targeted nursing staff, as they were the present consistently during the three checklist phases. Participants perceived these sessions as motivating.

End-users agreed that *PTB* made explicit information team members had normally communicated in silos rather than shared as a collective. Instances where checklist use had demonstrable impacts in averting potential adverse events likely increased motivation for sustained use of *PTB*. Participants considered the strategies within *PTB* helpful in embedding the checklist and described the proximal benefits:

*Improvements as a result of PTB and better communication overall. People are rationalising and communicating more…..* (RN, group)

However, participants acknowledged the consequences of patchy or limited compliance:

*Only when a clinical incident happens that staff say “thank God we did this” because it was documented that this was confirmed, and everyone in the room agreed that the specimens were confirmed and documented. It doesn’t impact people until something happens….* (RN, individual)

## Discussion

Using a theory-based evaluation aided explanation of factors that enabled and inhibited implementation of *PTB* and in identifying the generative mechanisms likely to enhance its sustainability. We triangulated the results from the NoMAD survey alongside semi-structured qualitative interviews to obtain a more comprehensive picture in the evaluation of the implementation of the *PTB* intervention in practice. Despite that there was some evidence of patchy implementation, the relative normalisation of *PTB* was sustained through flexible co-construction; a notion supported in May’s original model [12]. In coproduction there is value, and indeed necessity in blurring of the boundaries between clinical and academic staff in developing and implementing any practice-change interventions.

Our implementation team included a well-respected physician (surgeon) with influence as an opinion leader within the organisation. Because of this physician’s influence, the project team had the in-principle support of surgeons within the department. However, engagement of other physician groups was particularly challenging. Members of the implementation team invited all medical specialty groups to become involved during presentations, in emails, and staff meetings, with limited success. However, flexibility to co-create the intervention capitalising on the clinical experience of the clinical team mitigated to some extent, the effects of limited engagement in the work of implementation.

In this facility, while mandatory, checklist implementation was characterised by diffuse patterns of activity across the different surgical specialties, operationalising a best practice initiative that had been ‘enshrined’ in hospital policy. Notably, the workflow provided opportunities for nursing staff to participate more in *PTB* as they were present during all checklist phases, whereas, physicians were involved in other case or patient-related activates—curtailing their participation at that time. Therefore any intervention designed to improve consistency in checklist use, had to consider workflow. Clearly the complexity and workflow of surgery shapes the context in which individuals and teams work, and subsequently how health professionals interact with multifaceted interventions [8, 30].

Our findings suggest that the motivators for behaviour change in practice vary across professional groups, and are intrinsically linked to individuals’ professional identity and cultural resistance [31–33]. Normalisation of *PTB* was manifest among most nurses and some surgeons. The strong leadership roles assumed by several key senior nurses reflected program ‘champions’. On numerous occasions, nurses used their skills of negotiation to persuade and enrol others, particularly physicians, and organise or reorganise workflow workarounds. Program champions framed *PTB* as a requirement to improve team communications and an opportunity to clarify team members’ concerns. *PTB* had low coherence for physicians, who believed that the Sign-out phase did not make clinical sense. This culminated in low cognitive participation with physicians, especially anaesthetists, seeing little point to *PTB*. As such, physicians who struggled to see the need or value in yet another “redundant” practice, challenged nurses. On occasion, this impacted the extent to which nurses were able to implement the program, and in these instances, nurses used workarounds to achieve similar ends. Cultural resistance impeded the implementation of *PTB*, therefore hierarchical boundaries and traditions meant that not all physicians were willing to work with others in implementation. Clearly, given the hierarchical nature of surgery, physician commitment to implementation is imperative to sustain change [4, 8, 30, 34].

Normalisation of *PTB* in practice relied on the value ascribed to each phase of the SSC. For physicians, the consequences of omissions in verifying information as part of Timeout checks (i.e., just before knife-to-skin) carried heavy professional and financial penalties. Timeout checks represented the last defence against catastrophic errors, the most obvious being a wrong-site procedure [26, 27]. Sign-out checks (done prior to removal of drapes) confirm the procedure performed, instrument and swab counts, and plans for postoperative management. Nevertheless physicians saw no benefit in verifying this information as this section is not obviously linked to catastrophic events associated with the omission of Timeout, a notion identified in previous research [28]. Hence legitimation of Sign-out in practice was limited for physicians because of their scepticism of the evidence base, a finding highlighted elsewhere [16, 23, 25]. Physicians believed that the existing evidence in using Sign-out was insufficient to support its implementation as a safety initiative. From an implementation perspective, future research in this area should focus on developing targeted strategies to increase physician engagement in the process.

### Strengths and limitations

The strengths of this study are its longitudinal design and triangulated approach used to capture *what* surgical team members actually did and *how* they worked to embed *PTB* at two critical time points. A single site study and a 39% response rate while acceptable, limits generalisability. The potential for selection bias through convenience sampling is also a limitation. Additionally, physicians comprised only 19.2% of interview participants, so their views were likely underrepresented. While there was moderate buy-in from senior physicians during the developmental stage, the variation in spread likely contributed to its patchy implementation in some specialties.

Using a theory-driven approach to implementation is a strength of this study, but it also posed some challenges. NPT was useful to better understand and explain the social processes through which modifications to checklist implementation and sustained use could be evaluated. NPT, like any middle-range theory, does not claim to be a ‘theory of everything’ [35]. A challenge in its application was the overlapping nature of the four constructs, meaning that the qualitative data could be coded into more than one construct. We therefore made a decision to code data into more than one construct where relevant. Additionally, we occasionally found it difficult to be certain that we were categorising data into the ‘correct’ construct. There was also the potential for tension between using an abductive approach [36] while ensuring the data was not ‘forced’ into predefined constructs. Coding the data inductively using a thematic analysis before transposing it onto the constructs of NPT helped to address this since we first inductively coded and scrutinised all data for issues relating to implementation before applying NPT. These issues are consistent in the literature [35]. To mitigate this, we collaborated closely throughout the decision making process in coding the data.

Due to a small survey sample size, we were unable to undertake psychometric evaluation of the NoMADS tool. While the four NPT constructs demonstrated adequate internal consistency, Cronbach’s α for the construct *collective action* was slightly lower than the desired α 0.70. Recent psychometric testing of the NoMAD tool demonstrated a three-factor rather than a four factor structure [13], which may partly explain our results. A strength of this study is that the strategies used to embed *PTB* were implemented and tailored to the context by the key stakeholders. Coproduction and the ability to adapt complex interventions to context means that they are more likely to be sustained in practice [21, 22]. All interviews were conducted by the lead author who worked with implementation leaders to facilitate stakeholder engagement. Interviews were candid, surgical team participants did not feel obliged to report success, as evident from some of the transcripts.

## Conclusions

Programs such as *PTB* are often pragmatic in that implementation needs to respond to the day-to-day needs of the local context. As such, tailoring in any implementation effort is pivotal. Using the *NPT* model allowed us to uncover generative mechanisms to explain how the *PTB* program may lead to the continued normalisation of the checklist in practice. Flexible co-construction, workarounds and strong leadership are necessary ingredients for the successful sustainment of checklist use. However, further research is needed to identify strategies to engage physicians in the implementation process.

## Additional files

Additional file 1: NoMAD Survey tool, adapted from: May, C., Rapley, T., Mair, F.S., Treweek, S., Murray, E., Ballini, L., Macfarlane, A. Girling, M. and Finch, T.L. (2015) Normalization Process Theory On-line Users’ Manual, Toolkit and NoMAD instrument. Available from: http://www.normalizationprocess.org/nomad-study/. (DOC 215 kb)

Additional file 2: Interview guide questions. (DOC 52 kb)

## Abbreviations

NoMAD: NOrmalization MeAsure Development
NPT: Normalisation Process Theory
PTB: Pass The Baton
SSC: Surgical Safety Checklist
WHO: World Health Organisation
