# Supplementary material for: Using normalisation process theory to evaluate the implementation of a complex intervention to embed the surgical safety checklist
Source: BMC Health Serv Res. 2018 Mar 9;18:170. doi: 10.1186/s12913-018-2973-5 (PMC5845378; doi:10.1186/s12913-018-2973-5)
Supplement: Supplementary file 1 — NoMAD Survey tool, adapted from: May, C., Rapley, T., Mair, F.S., Treweek, S., Murray, E., Ballini, L., Macfarlane, A. Girling, M. and Finch, T.L. (2015) Normalization Process Theory On-line Users’ Manual, Toolkit and NoMAD instrument. Available from: http://www.normalizationprocess.org/nomad-study/. (DOC 215 kb) [file 12913_2018_2973_MOESM1_ESM.docx]

| ***Additional file 1: NoMAD Survey***  **Survey Instructions** | 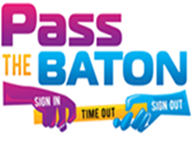 |
| --- | --- |

**This survey is designed to help get a better understanding of how to incorporate work process strategies into everyday practice.**

This survey asks questions about the implementation of *Pass The Baton.* We understand that people involved with *Pass The Baton* have different roles, and that people may have more than one role.

From the statements below please choose an option that best describes ***your main role*** in relation to *Pass The Baton*:

I am involved in managing or overseeing *Pass The Baton*

I am involved in delivering *Pass The Baton*

I am a participant/recipient of *Pass The Baton*

For this survey, please answer all the statements from the perspective of this role. Depending on your role or responsibilities in *Pass The Baton,* some statements may be more relevant than others.

This survey is in three parts. Part A asks some brief questions about yourself and your role. Part B includes three general questions about *Pass The Baton.* Part C contains a set of more detailed questions about *Pass The Baton.* For each statement in Part C, there is the option to agree or disagree with what is being asked **(OPTION A).** However, if you feel that the statement is not relevant to you, there are also options to tell us why **(OPTION B).**

Please take time to decide which answer **best suits your experience for each statement and tick the appropriate circle.**

| **PART A: Demographics** |  |
| --- | --- |
| 1. What is your gender?  Male ^0^  Female ^1^ |  |
| 1. For how long have you practiced in your current clinical role?   _________ Years _________ months |  |
| 1. What are you primarily employed as?   ^1^  Enrolled Nurse_­­­_  ^2^  Registered Nurse  ^3^  Clinical Nurse / Clinical Nurse Specialist  ^4^  NUM / Nurse Educator  ^5^  RMO  ^6^  Registrar  ^7^  Consultant  ^8^  Other (please state beside) _______________________________ |  |
| 1. What is your highest qualification?   ^1^  Hospital certificate  ^2^  Diploma  ^3^  Degree  ^4^  Postgraduate Certificate  ^5^  Postgraduate Diploma  ^6^  Masters  ^7^  PhD |  |
| 1. What specialty do you mainly work in?   ^1^  Anaesthetics  ^2^  Surgery |  |

| **PART B: General questions about *Pass The Baton*** |  |
| --- | --- |
| Please refer to following scale for Question 1:   \| 1 \| 2 \| \| 3 \| 4 \| \| 5 \| 6 \| \| 7 \| 8 \| 9 \| \| 10 \| \| --- \| --- \| --- \| --- \| --- \| --- \| --- \| --- \| --- \| --- \| --- \| --- \| --- \| --- \| \| **Still feels very new** \|  \|  \|  \|  \|  \|  \|  \|  \|  \|  \|  \|  \| **Feels completely familiar** \| \| |  |
| 1. When you use *Pass The Baton,* how familiar does it feel? |  |
| _1_ _2_ _3_ _4_ _5_ _6_ _7_ _8_ _9_ _10_ |  |

| \| Please refer to the following scale for Questions 2 and 3: \| \| --- \| \| \| 1 \| 2 \| \| 3 \| 4 \| \| 5 \| 6 \| \| 7 \| 8 \| 9 \| \| 10 \| \| --- \| --- \| --- \| --- \| --- \| --- \| --- \| --- \| --- \| --- \| --- \| --- \| --- \| --- \| \| **Not at all** \|  \|  \|  \|  \|  \| **Somewhat** \|  \|  \|  \|  \|  \|  \| **Completely** \| \| \| \|  \| |
| --- | --- | --- | --- | --- | --- | --- | --- | --- | --- | --- | --- | --- | --- | --- | --- | --- | --- | --- | --- | --- | --- | --- | --- | --- | --- | --- | --- | --- | --- | --- | --- | --- |
|  |
| \| 1. Do you feel *Pass The Baton* is currently a normal part of your work? \| \| \| \| \| \| \| \| \| \| \|  \| \| --- \| --- \| --- \| --- \| --- \| --- \| --- \| --- \| --- \| --- \| --- \| --- \| \| _1_ _2_ _3_ _4_ _5_ _6_ _7_ _8_ _9_ _10_ \| \| \| \| \| \| \| \| \| \| \| \| \| 1. Do you feel *Pass The Baton* will become part of your work? \| \| \| \| \| \| \| \| \| \| \| \| \| _1_ _2_ _3_ _4_ _5_ _6_ _7_ _8_ _9_ _10_ \| \| \| \| \| \| \| \| \| \| \| \| \| **PART C: Detailed questions about *Pass The Baton*** \| \| \| \| \| \| \| \| \| \| \| \| \| **For each of the statements in Sections C1, C2, C3 and C4, please select an answer that best suits your experience using Option A. If the statement is not relevant to you please select an answer from Option B.** \| \| \| \| \| \| \| \| \| \|  \| \| \|  \| **OPTION A** \| \| \| \| \|  \| **OPTION B** \| \| \| \| \| \| \| **Section C1** \| **Strongly Agree** \| **Agree** \| **Neither agree nor disagree** \| **Disagree** \| **Strongly disagree** \|  \| **Not relevant to my role** \| **Not relevant at this stage** \| **Not relevant to *Pass The Baton*** \| \| \| \| \| 1. I can see how *Pass The Baton* differs from usual ways of working \|  \|  \|  \|  \|  \|  \|  \|  \|  \| \| \| \| \| 1. Staff in this organisation have shared understanding of the purpose of *Pass The Baton* \|  \|  \|  \|  \|  \|  \|  \|  \|  \| \| \| \| \| 1. I understand how *Pass The Baton* affects the nature of my own work \|  \|  \|  \|  \|  \|  \|  \|  \|  \| \| \| \| \| 1. I can see the value of *Pass The Baton* for my work \|  \|  \|  \|  \|  \|  \|  \|  \|  \| \| \| \|  \|  \| **OPTION A** \| \| \| \| \|  \| **OPTION B** \| \| \| \| --- \| --- \| --- \| --- \| --- \| --- \| --- \| --- \| --- \| --- \| \| **Section C2** \| **Strongly Agree** \| **Agree** \| **Neither agree nor disagree** \| **Disagree** \| **Strongly disagree** \|  \| **Not relevant to my role** \| **Not relevant at this stage** \| **Not relevant to *Pass The Baton*** \| \| 1. There are key people who drive *Pass The Baton* forward and get others involved \|  \|  \|  \|  \|  \|  \|  \|  \|  \| \| 1. I believe that participating in *Pass The Baton* is a legitimate part of my role \|  \|  \|  \|  \|  \|  \|  \|  \|  \| \| 1. I’m open to working with colleagues in new ways to use *Pass The Baton* \|  \|  \|  \|  \|  \|  \|  \|  \|  \| \| 1. I will continue to support *Pass The Baton* \|  \|  \|  \|  \|  \|  \|  \|  \|  \| |

|  | **OPTION A** | | | | |  | **OPTION B** | | |
| --- | --- | --- | --- | --- | --- | --- | --- | --- | --- |
| **Section C3** | **Strongly Agree** | **Agree** | **Neither agree nor disagree** | **Disagree** | **Strongly disagree** |  | **Not relevant to my role** | **Not relevant at this stage** | **Not relevant to *Pass The Baton*** |
| 1. I can easily integrate *Pass The Baton* into my existing work |  |  |  |  |  |  |  |  |  |
| 1. *Pass The Baton* disrupts working relationships |  |  |  |  |  |  |  |  |  |
| 1. I have confidence in other people’s ability to use *Pass The Baton* |  |  |  |  |  |  |  |  |  |
| 1. Work is assigned to those with skills appropriate to *Pass The Baton* |  |  |  |  |  |  |  |  |  |
| 1. Sufficient training is provided to enable staff to implement *Pass The Baton* |  |  |  |  |  |  |  |  |  |
| 1. Sufficient resources are available to support *Pass The Baton* |  |  |  |  |  |  |  |  |  |
| 1. Management adequately supports *Pass The Baton* |  |  |  |  |  |  |  |  |  |

|  | **OPTION A** | | | | |  | **OPTION B** | | |
| --- | --- | --- | --- | --- | --- | --- | --- | --- | --- |
| **Section C4** | **Strongly Agree** | **Agree** | **Neither agree nor disagree** | **Disagree** | **Strongly disagree** |  | **Not relevant to my role** | **Not relevant at this stage** | **Not relevant to *Pass The Baton*** |
| 1. I am aware of reports about the effects of *Pass The Baton* |  |  |  |  |  |  |  |  |  |
| 1. The staff agree that *Pass The Baton* is worthwhile |  |  |  |  |  |  |  |  |  |
| 1. I value the effects that *Pass The Baton* has had on my work |  |  |  |  |  |  |  |  |  |
| 1. Feedback about *Pass The Baton* can be used to improve it in the future |  |  |  |  |  |  |  |  |  |
| 1. I can modify how I work with *Pass The Baton* |  |  |  |  |  |  |  |  |  |

**Thank you for completing our survey**

Note: Survey was adapted from: *May, C., Rapley, T., Mair, F.S., Treweek, S., Murray, E., Ballini, L., Macfarlane, A. Girling, M. and Finch, T.L. (2015) Normalization Process Theory On-line Users’ Manual, Toolkit and NoMAD instrument. Available from:* [*http://www.normalizationprocess.org*](http://www.normalizationprocess.org)
