# Supplementary material for: Using normalisation process theory to evaluate the implementation of a complex intervention to embed the surgical safety checklist
Source: BMC Health Serv Res. 2018 Mar 9;18:170. doi: 10.1186/s12913-018-2973-5 (PMC5845378; doi:10.1186/s12913-018-2973-5)
Supplement: Supplementary file 2 — Interview guide questions. (DOC 52 kb) [file 12913_2018_2973_MOESM2_ESM.docx]

***Additional file 2: Interview guide questions***

1. How is pass the baton [PTB] different from what you are already doing when using the checklist?
2. Do members of the surgical team agree on the intent and benefit of using PTB?
3. Do team members understand how PTB affects their day-to-day roles, tasks and responsibilities?
4. Is there coherence in the tasks and responsibilities that comprise PTB?
5. Do you think that you have had enough training to use PTB?
6. Do you think other team members are confident in using PTB?
7. What do you do differently as a result of using PTB?
8. In what ways do you think that PTB could be improved?
9. What components and/or actions about PTB would you change? Why?
10. Is PTB supported in the OR department? In what ways is PTB supported or not supported in the department?
